# An East End Hospital

**Published:** 1924-06

**Authors:** 


### AN EAST END HOSPITAL.

In that delightful building, the Grocers' Hall, in Princes Street, E.C., on May 7, the City of London Hospital for Diseases of the Heart and Lungs (formerly the London Chest Hospital) held the annual meeting of the Court of Governors.

### Coming Developments.

In submitting the report and accounts for the year, the chairman, Sir Alexander Kaye Butterworth, regretted that the figures showed a substantial excess of expenditure over receipts, happily a thing which seldom occurs. He then drew attention to the resolution of the Committee to carry out an extensive series of improvements in the hospital and its equipment, in spite of the possible objection that this is a bad time for incurring expenses which might be avoided. These same improvements, he said, were found to be necessary ten years ago and the work was started then, but, owing to the outbreak of the war, had to be postponed. Another appeal was started in 1919, but circumstances forced the Committee to abandon it; now, however, they had come to the conclusion that they could not afford to wait any longer and a new appeal is to be launched. The improvements and alterations to be undertaken consist of the completion of the electric lighting, the construction of a new sanitary annexe and a new surgical block at the hospital, while, at the Saunderton sanatorium, an extension of the buildings is to be put in hand. A loan had to be repaid to the bank for the building of the road to, and for the installing of acetylene gas in, the sanatorium.

### The Cost and the Need.

For all this the estimated cost was £30,000, and in order to raise that amount a special appeal was to be inaugurated, by permission of the Lord Mayor, at a luncheon at the Mansion House on June 2. The President, the Duke of Connaught, had promised to take the chair and he would be supported by a distinguished gathering of peers and representative City men. The chairman laid stress on the fact that while the alterations at the hospital were needed in order to keep it up to date and ensure the continued efficiency of its work, they would not involve any substantial increase in the working costs—except, perhaps, in the sanatorium. They would enable the hospital to be run more economically, and he made a strong appeal to all who are interested in the maintenance of the voluntary hospital system, especially those in the city from which the hospital takes its name, to support the appeal. The treasurer, Sir George Wyatt Truscott, the Committee, and the auditors were then re-elected for the coming year. Sir George Truscott proposed a vote of thanks to the medical staff, which was seconded by Mr. F. Cornish. Votes of thanks were also given to the Grocers' Company for the use of their hall, to the auditors, and to the chairman for his most valuable services during the past year.

